# Plasma Fatty Acid Ratios Affect Blood Gene Expression Profiles - A Cross-Sectional Study of the Norwegian Women and Cancer Post-Genome Cohort

**DOI:** 10.1371/journal.pone.0067270

**Published:** 2013-06-25

**Authors:** Karina Standahl Olsen, Christopher Fenton, Livar Frøyland, Marit Waaseth, Ruth H. Paulssen, Eiliv Lund

**Affiliations:** 1 Department of Community Medicine, University of Tromsø, Tromsø, Norway; 2 Department of Clinical Medicine, University of Tromsø, Tromsø, Norway; 3 National Institute of Nutrition and Seafood Research (NIFES), Bergen, Norway; 4 Department of Pharmacy, University of Tromsø, Tromsø, Norway; Max Delbrueck Center for Molecular Medicine, Germany

## Abstract

High blood concentrations of n-6 fatty acids (FAs) relative to n-3 FAs may lead to a “physiological switch” towards permanent low-grade inflammation, potentially influencing the onset of cardiovascular and inflammatory diseases, as well as cancer. To explore the potential effects of FA ratios prior to disease onset, we measured blood gene expression profiles and plasma FA ratios (linoleic acid/alpha-linolenic acid, LA/ALA; arachidonic acid/eicosapentaenoic acid, AA/EPA; and total n-6/n-3) in a cross-section of middle-aged Norwegian women (n = 227). After arranging samples from the highest values to the lowest for all three FA ratios (LA/ALA, AA/EPA and total n-6/n-3), the highest and lowest deciles of samples were compared. Differences in gene expression profiles were assessed by single-gene and pathway-level analyses. The LA/ALA ratio had the largest impact on gene expression profiles, with 135 differentially expressed genes, followed by the total n-6/n-3 ratio (125 genes) and the AA/EPA ratio (72 genes). All FA ratios were associated with genes related to immune processes, with a tendency for increased pro-inflammatory signaling in the highest FA ratio deciles. Lipid metabolism related to peroxisome proliferator-activated receptor γ (PPARγ) signaling was modified, with possible implications for foam cell formation and development of cardiovascular diseases. We identified higher expression levels of several autophagy marker genes, mainly in the lowest LA/ALA decile. This finding may point to the regulation of autophagy as a novel aspect of FA biology which warrants further study. Lastly, all FA ratios were associated with gene sets that included targets of specific microRNAs, and gene sets containing common promoter motifs that did not match any known transcription factors. We conclude that plasma FA ratios are associated with differences in blood gene expression profiles in this free-living population, and that affected genes and pathways may influence the onset and progression of disease.

## Introduction

The type and amount of fatty acids (FAs) in a person’s diet determine the relative amounts of FAs in the tissues of the body, and may influence the pathogenesis of cardiovascular and inflammatory diseases, as well as cancer [1]–[3]. A multitude of mechanisms have been shown to influence disease pathogenesis, including lipid metabolism and inflammation. To study the complex molecular mechanisms involved in the association between nutritional factors and multifactorial diseases, high-throughput technologies like transcriptomics are increasingly being used.

The importance of dietary fat is mirrored in the complexity of FA metabolism. After ingestion, or following lipolysis in adipose tissue, FAs enter the blood stream either esterified in lipoproteins or non-esterified bound to albumin. FAs are transported into cells throughout the body, where they may be degraded by (per-) oxidation, stored as triglycerides, or incorporated into the phospholipids of cellular membranes. Membrane phospholipids can be modified into lipid mediators such as inositol triphosphate (IP_3_), and FAs residing in the cellular membranes can be mobilized through the action of phospholipases and undergo modifications to yield a variety of immunoactive eicosanoids. Furthermore, FAs are potent regulators of gene expression via receptors like the peroxisome proliferator-activated receptors (PPARs) [4], [5]. Collectively, PPARs and their binding partners, retinoid X receptors (RXR), regulate cellular and physiological processes including FA metabolism, cellular stress, and inflammation.

A key aspect of FA biology is the specificity inferred by the FA structure, as well as the fact that dietary sources differ in the FAs they contain. Two long-chain polyunsaturated FAs (PUFAs) are essential to humans and must be obtained from the diet: linoleic acid (LA, 18∶2 n-6) and alpha-linolenic acid (ALA, 18∶3 n-3), both of which are derived mainly from plant oils. LA is metabolized to arachidonic acid (AA, 20∶4 n-6), whereas ALA is converted to eicosapentaenoic acid (EPA, 20∶5 n-3) and docosahexaenoic acid (DHA, 22∶6 n-3). This metabolism of PUFAs is extremely limited in humans [6]. In addition, mammals cannot convert n-6 FAs to n-3 FAs [7], making the two classes metabolically distinct. n-6 and n-3 PUFAs compete for the same enzyme systems, so that the conversion of ALA to EPA and DHA is further reduced in individuals who adhere to a Western diet, due to the high intake of LA. Hence, EPA and DHA are mainly provided from dietary marine sources.

It has been suggested that n-6 FAs are pro-inflammatory, whereas n-3 FAs are much less so, and sometimes even anti-inflammatory. The ratio of n-6/n-3 FAs has been implicated in the pathogenesis of conditions like inflammatory and cardiovascular diseases, as well as cancers of the breast, prostate and colon [1], [8], [9]. A dietary n-6/n-3 ratio of 1 may be considered healthy, corresponding to a 50% n-3 FA content in tissues, but the typical Western diet provides a considerably higher n-6/n-3 ratio [10]. However, a relatively high n-3 intake and low n-6/n-3 ratio is observed in Norwegians, who consume high amounts of fish, fish products and fish oil supplements compared to other Western populations [11], [12]. Effects of dietary intervention studies to reduce the n-6/n-3 ratio in healthy subjects include a reduced number of platelets and leukocytes [13], and beneficial modulation of metabolic and inflammatory markers [14], [15]. The involvement of inflammatory mechanisms in the association between n-6/n-3 ratio and disease has led to the hypothesis that a high n-6/n-3 ratio may cause a “physiological switch” towards a permanent state of low-grade inflammation, which may be detrimental to several aspects of human health [1], [16].

Despite the overarching hypothesis of a “physiological inflammatory switch”, genome-wide transcriptomics studies exploring the potential effects of the n-6/n-3 ratio in the general population are scarce. However, some intervention studies have used FA supplementation to investigate the effects on gene expression levels. One targeted study of healthy adults showed reduced expression of selected signal transduction genes and pro-inflammatory cytokines after a 40% reduction of the n-6/n-3 ratio [17]. In addition, short and long-term effects of FA supplementation on genome-wide transcription in blood cells has been investigated [18]. Postprandial gene expression changes were found in peripheral blood mononuclear cells (PBMCs) after a DHA-rich meal, including up-regulation of stress genes and down-regulation of lipid metabolism [19]. After long-term supplementation (26 weeks) with EPA/DHA in an elderly population, PBMC gene expression levels related to inflammatory and atherogenic pathways were decreased [5].

Taken together, molecular and epidemiological studies indicate the importance of the n-6/n-3 ratio in relation to the prevention of inflammatory and cardiovascular diseases, as well as cancer. However, the use of the n-6/n-3 ratio to monitor and modify risk factors related to human disease is not without controversy [20]. One important objection stems from the fact that the total n-6/n-3 ratio, which is often used in epidemiological studies, does not distinguish between different types of PUFAs, e.g. LA/ALA or AA/EPA ratios. Furthermore, the extrapolation of the positive effects of FA ratios on disease, to their potential effects during disease onset, has not been extensively evaluated. With the aim of exploring the potential mechanisms of FA ratios in disease prevention, we used data from the Norwegian Women and Cancer (NOWAC) Post-genome Cohort [21] to perform a cross-sectional analysis of middle-aged Norwegian women. We grouped the study population according to three plasma FA ratios (LA/ALA, AA/EPA and total n-6/n-3), and identified differences in blood gene expression profiles by comparing the highest versus the lowest FA ratio deciles. To our knowledge, the present study is the first to explore the genome-wide transcriptomic effects of FA ratios in a representative cohort.

## Materials and Methods

### Study Population and Materials

The NOWAC Study [22] and the NOWAC Post-genome Cohort [21] have been described in detail elsewhere. Briefly, the Post-genome Cohort consists of 50 000 women born between 1943 and 1957. Invited women were asked to visit their general physician to have blood collected using a kit which they received by mail, and to answer a two-page questionnaire on anthropometric and lifestyle parameters (including use of dietary supplements, medication, and menstrual status). Blood samples and questionnaires were returned by mail to the NOWAC study center. A total of 500 women were randomly selected for the present study, 444 of whom returned blood samples and questionnaires during 2005 (response rate 89%). All women included in our study population successfully returned a citrate-buffered blood sample, and a PAXgene blood RNA collection tube, which conserves the RNA profile of all circulating cells (Preanalytix, Qiagen, Hilden, Germany). Blood samples were required to reach the study center and be frozen no more than 3 days after blood collection. Furthermore, all included women were required to be postmenopausal according to self-report and plasma hormone levels (99 excluded) [23]. Medication was classified into the following categories: diabetes, non-steroidal anti-inflammatory drugs (NSAIDs), statins, cardiovascular medication (diuretics, adrenergic beta antagonists, calcium channel blockers, renin-angiotensin system inhibitors (including combinations with diuretics) and other anti-hypertensives), and immune system-related medication. Based on this classification, 30 women taking diabetes medication, statins, and immune system-related medication were excluded. In addition, two women using anti-thrombotics, and one using hydroxychloroquine were excluded from the study. Women were not required to fast before blood collection, but those who had not eaten for 12 h or more were defined as fasting. Women who had smoked during the week before blood collection were defined as smokers, and those who had taken n-3 capsules/oils and/or cod liver oil the week before blood collection were defined as users of n-3 supplements.

### Ethics Statement

The NOWAC Post-genome Cohort study was approved by the Regional Committee for Medical Research Ethics and the Norwegian Data Inspectorate. All participants gave written informed consent.

### RNA Extraction and Gene Expression Profiling

Details of experimental procedures have been described elsewhere [24]. Total RNA was isolated using the PAXgene Blood RNA Isolation Kit (Preanalytix, Qiagen, Hilden, Germany), according to the manufacturer’s protocol. RNA quantity, quality and integrity were assessed using the NanoDrop ND1000 spectrophotometer (Thermo Scientific, Wilmington, Delaware, USA) and the Experion automated electrophoresis system (BioRad Laboratories, Hemel Hempstead, UK); 39 samples were excluded due to low RNA quality or quantity. No globin reduction method was used, in line with our previous finding that showed no major benefit from reduction of globin RNA for the microarray platform employed in the present study [25]. Gene expression levels were measured using the Human Genome Survey Microarray V2.0 (Applied Biosystems, ABI, Life Technologies, Carlsbad, California, USA). The microarray contains 32 878 probes for the interrogation of 29 098 transcripts. ABI Expression System software was used to extract signal intensities and signal/noise ratios, and for flagging of spots. Experimental details and required data files were submitted to Gene Expression Omnibus (ww.ncbi.nlm.nih.gov/geo), under accession number GSE15289.

### Microarray Data Preprocessing and Normalization

The Bioconductor/R statistical package was used for microarray data preprocessing (script available upon request). Standard ABI exclusion criteria were applied, removing probes with a signal/noise ratio of less than 3, or those that were flagged as irregular by the scanner. There were 10 784 probes that met the selection criteria, and these were included in the gene expression matrix. Missing values were imputed using the k-nearest neighbor method [26]. Arrays that displayed an irregular intensity pattern in the built-in controls were removed (n = 3). The gene expression data set was normalized using a quantile and control-based normalization approach [27] (script available upon request). Probes with very low variability (<0.1), or very low average log2 signal (<9.5), were removed.

### FA Measurements

The National Institute of Nutrition and Seafood Research analyzed 34 unique FAs in citrate-buffered plasma samples, using rapid gas chromatography [28]. Failed measurements caused eight samples to be excluded. FA ratios were calculated from the mass of each FA.

### Statistical Analysis

The final number of included women was 227. For this explorative analysis, gene expression matrices were generated by arranging samples (columns) from highest to lowest values for the three FA ratios considered (LA/ALA, AA/EPA and total n-6/n-3). Within each matrix only the highest and lowest decile of samples were compared (decile n = 23). Potential confounders were evaluated by comparing the highest and lowest deciles using independent sample t-tests with two-tailed p-values, Mann-Whitney U tests, and Chi square tests (SPSS Statistics 19, IBM, Armonk, New York), with p<0.01 as the significance threshold. There was a small, but statistically significant age difference among women in the highest versus the lowest AA/EPA and total n-6/n-3 deciles. Changes in lipid metabolism during menopausal transition have been well characterized, but as of yet, time since menopause has not been reported to influence lipid profiles [29]. Because all of our study women were postmenopausal, we chose not to adjust for age in the analyses of gene expression profiles. T-tests in R, and Benjamini-Hochberg corrected p-values were used to evaluate differential gene expression between the highest and the lowest LA/ALA, AA/EPA and total n-6/n-3 deciles. Functional annotations of single genes were explored using www.genecards.org and The Database for Annotation, Visualization and Integrated Discovery v. 6.7 [30]. Lists of differentially expressed genes were evaluated for potential overlap with previously reported gene sets using the Molecular Signatures Database (MSigDB) v. 3.0 (www.broadinstitute.org/gsea/msigdb) [31], limited to collections C2 CP (Kyoto Encyclopedia of Genes and Genomes Pathway Database, KEGG, Biocarta, Reactome), C3 (microRNA, miRNA, targets, transcription factor targets), and C5 (Gene Ontology, GO). Gene Set Enrichment Analysis (GSEA) [31] in R was used for functional interpretation based on gene sets from KEGG, GO and Panther. False discovery rate (FDR) <25% and nominal p<0.05 were used as significance thresholds in GSEA.

## Results

Our study population (n = 227) was slightly overweight, and one-quarter of them were smokers (Table 1). A majority of the study women had taken n-3 supplements, in concordance with previous findings in the Norwegian population [12]. Nevertheless, the population mean of all FA ratios was dominated by n-6 FAs. The mean LA/ALA ratio was approximately 50, mean AA/EPA ratio was approximately 4, and mean total n-6/n-3 ratio was approximately 6. The highest and lowest FA ratio deciles included 23 women each, and their characteristics are presented in Table 2 and Supplementary Table S1. There were no significant differences in body mass index, smoking, fasting and medication use between comparison groups (p<0.01). There were no significant differences in main characteristics between the highest and lowest LA/ALA deciles. When comparing the highest versus the lowest AA/EPA deciles, the lowest decile was slightly older and had a higher frequency of n-3 supplement use. The same pattern of age and supplement use was present in the comparison of the highest versus the lowest total n-6/n-3 deciles. In the study population as a whole, users of n-3 supplements were slightly older than non-users (2 years older, p<0.01, data not shown), but the age difference was statistically significant. The frequent use of supplements may be related to the AA/EPA ratio of the lowest AA/EPA decile and the lowest total n-6/n-3 decile (AA/EPA ratio 1.3 and 1.5, respectively), which may be regarded as balanced (Table 2). There were no significant differences between any of the FA ratio deciles regarding technical variables (time elapsed between blood collection and freezing of the blood sample, date of RNA extraction, or microarray lot numbers, data not shown).

**Table 1:** Characteristics of the 227 women in the study population^a^.

Characteristic	Mean or frequency
Age	55.6±3.5
BMI	25.5±4.4
Smoking	59 (26%)
Fasting	13 (7%)
Time since meal (h)	2.7±3.7
n-3 supplements	130 (59%)
NSAIDs	20 (9%)
Cardiovascular medication	30 (13%)
HRT	39 (17%)
LA/ALA	51.9±17.8
AA/EPA	4.3±2.8
n-6/n-3	6.1±2.0

a Format for age, BMI, time since meal, and FA ratios: mean (standard deviation). Format for smoking, fasting, supplements and medication: frequency (percent). Missing: BMI: 3, smoking: 1, fasting: 27, n-3 supplements: 5.

**Abbreviations:** AA: arachidonic acid, ALA: alpha-linolenic acid, BMI: body mass index, EPA: eicosapentaenoic acid, h: hours, HRT: hormone replacement therapy, LA: linoleic acid, n-3 supplements: any combination of n-3 capsules/oils, cod liver oil or both, NSAIDs: non-steroidal anti-inflammatory drugs.

**Table 2:** Characteristics of the highest and lowest fatty acid ratio deciles^a^.

	LA/ALA ratio			AA/EPA ratio			n6/n3 ratio		
	Highest decile	Lowest decile	p	Highest decile	Lowest decile	p	Highest decile	Lowest decile	p
Age	55±3.7	55±3.3	0.97	54±3.5	57±3.0	<0.01	53±3.3	57±3.2	<0.01
BMI	24±2.8	26±3.6	0.17	24±4.0	23±2.5	0.4	23±3.8	24±4.0	0.47
Smoking	8 (35%)	5 (22%)	0.51	8 (36%)	2 (9%)	0.04	8 (36%)	3 (13%)	0.14
Fasting	3 (16%)	1 (5%)	0.33	2 (10%)	0	0.23	0	0	NA
Time since meal (h)	3.7±5.1	1.9±2.6	0.24	3.2±4.1	1.9±1.7	0.18	1.7±1.2	2.2±2.4	0.72
n-3 suppl.	12 (52%)	14 (61%)	0.77	3 (14%)	21 (91%)	<0.01	4 (18%)	22 (96%)	<0.01
NSAIDs	2 (9%)	0	0.49	4 (17%)	1 (4%)	0.35	5 (22%)	3 (13%)	0.70
Cardiovascular medication	3 (13%)	5 (22%)	0.70	4 (17%)	0	0.11	2 (9%)	1 (4%)	1.00
HRT	5 (22%)	2 (9%)	0.41	9 (17%)	3 (13%)	0.09	8 (35%)	3 (13%)	0.07
LA/ALA	86.8±14.2	26±5.0	<0.01	61.0±23.9	46.4±16.4	0.02	62.1±23.9	45.5±15.0	0.01
AA/EPA	4.9±3.1	3.2±1.9	0.02	10.4±2.8	1.3±0.2	<0.01	9.2±2.8	1.5±0.5	<0.01
n-6/n-3	6.9±2.3	5.0±1.6	<0.01	9.5±1.8	3.4±0.8	<0.01	10.0±1.4	3.1±0.5	<0.01

a Decile n = 23. Subgroups may not total 227 due to missing values.

**Abbreviations:** AA: arachidonic acid, ALA: alpha-linolenic acid, BMI: body mass index, EPA: eicosapentaenoic acid, h: hours, HRT: hormone replacement therapy, LA: linoleic acid, n-3 suppl.: any combination of n-3 capsules/oils, cod liver oil or both, NSAIDs: non-steroidal anti-inflammatory drugs.

The LA/ALA ratio was associated with the highest number of differentially expressed genes (Table 3, t-tests, adjusted p≤0.01, 315 genes), followed by the total n-6/n-3 ratio (125 genes), and the AA/EPA ratio (72 genes). We present the top 10 up- and down-regulated genes (Table 4–6), and complete gene lists are available (Supplemental Tables S2–S4). All tables contain gene symbols and corresponding gene names. Gene expression profiles according to AA/EPA and total n-6/n-3 ratios overlapped slightly more than the gene expression profile according to LA/ALA ratio, and no genes were shared across all three ratios (Supplemental Figure S1). As often seen when studying human complex tissues in a non-experimental setting, the biological variation between individuals was large and the magnitude of detectable gene expression changes was small: no transcripts changed more than approximately 1.4-fold between groups. Differentially expressed genes resulting from the comparison of the highest versus the lowest LA/ALA deciles included inflammatory markers like receptor subunits for IL1 and IL10 (IL1R2, IL10RB), chemokines (CCL7, CCL24), and TLR8 (Table 4 and Supplemental Table S2). Key FA metabolism regulators were differentially expressed, including PPARγ binding partners. Interestingly, genes related to autophagy were more highly expressed in the lowest LA/ALA decile, including KIAA0831 and OPTN. The list of differentially expressed genes overlapped with 50 gene sets in MSigDB belonging to two major categories (Table 7 and Supplemental Table S5): one category involving immune processes, with up to 20% overlap (e.g. the IL10 pathway, NO_2_-dependent IL12 pathway, and Systemic Lupus Erythematosus pathway), and one related to function, packaging, maintenance and repair of DNA and RNA (e.g. Reactome unwinding of DNA, 18% overlap). A significant overlap with gene sets corresponding to targets of two miRNAs was identified, as well as with three gene sets with common promoter motifs. However, neither motif matched any known transcription factors. According to GSEA (Table 8), the Jak/STAT pathway was positively enriched in the highest LA/ALA decile.

**Table 3:** Number of differentially expressed genes (t-tests, p≤0.01).

FA ratio	Differentially expressed	Up-regulated	Down-regulated
LA/ALA	315	168	147
AA/EPA	72	35	37
n-6/n-3	125	46	79

**Abbreviations:** AA: arachidonic acid, ALA: alpha-linolenic acid, EPA: eicosapentaenoic acid, FA: fatty acid, LA: linoleic acid.

**Table 4:** Top 10 up- and down-regulated genes associated with LA/ALA ratio (p≤0.01)^a^.

Gene symbol	Gene name	ABI probe ID	Entrez gene ID	Mean difference
HDC	Histidine decarboxylase	194873	3067	1.41
CPA3	Carboxypeptidase A3 (mast cell)	100989	1359	1.36
TCHHL1	Trichohyalin-like 1 (basalin, S100 calcium-binding protein A17)	132590	126637	1.20
MYBPHL	Myosin binding protein H-like	201601	343263	1.18
FcER1B	FC epsilon receptor I beta-chain (MS4A2)	182643	2206	1.00
ANPEP	Alanyl (membrane) aminopeptidase (CD13)	102558	290	0.64
GLT1D1	Glycosyltransferase 1 domain-containing protein 1	185381	144423	0.63
ZNF558	Zinc finger protein 558	124882	148156	0.63
SLC22A9	Solute carrier family 22 (organic anion/cation transporter) 9	206544	114571	0.62
FABP4	Fatty acid binding protein 4, adipocyte	150137	2167	0.61
IFIT1L	Interferon-induced protein with tetratricopeptide repeats 1B	112888	439996	−0.75
MINPP1	Multiple inositol polyphosphate phosphatase 1	188640	9562	−0.70
OR2W3	Olfactory receptor 2W3	702434	343171	−0.70
FAM63B	Family with sequence similarity 63, member B	161432	54629	−0.69
GFI1	Growth factor independent 1 transcription repressor (ZNF163)	121984	2672	−0.69
MATR3	Matrin 3	229698	9782	−0.66
SNCA	Synuclein, alpha (non A4 comp. of amyloid precursor)	170285	6622	−0.63
MYBL1	V-myb myeloblastosis viral oncogene homolog (avian)-like 1	207803	4603	−0.63
NR2C2AP	Nuclear receptor 2C2-associated protein	211955	126382	−0.59
KLRG1	Killer cell lectin-like receptor subfamily G, member 1	188801	10219	−0.58

a Complete lists of differentially expressed genes (p≤0.01) are given in Supplemental Table S2.

**Abbreviations:** ABI: Applied Biosystems, ALA: alpha-linolenic acid, LA: linoleic acid.

**Table 5:** Top 10 up- and down-regulated genes associated with AA/EPA ratio (p≤0.01)^a^.

Gene symbol	Gene name	ABI probe ID	Entrez gene ID	Mean difference
ABCB9	ATP-binding cassette, sub-family B (MDR/TAP), member 9	155893	23457	0.68
ZNF354A	Zinc finger protein eZNF (transcription factor 17)	214609	6940	0.64
ENTPD4	Ectonucleoside triphosphate diphosphohydrolase 4 (LYSAL1)	226738	9583	0.56
ZCCHC2	Zinc finger, CCHC domain-containing protein 2	154456	54877	0.51
RNMTL1	RNA methyltransferase-like protein 1	135382	55178	0.51
GRIN2C	Glutamate receptor, ionotropic, N-methyl D-aspartate 2C	194729	2905	0.47
PODNL1	Podocan-like 1	146598	79883	0.47
CCL24	Chemokine (C-C motif) ligand 24	128049	6369	0.44
RNF157	Ring finger protein 157	212544	114804	0.40
C20orf26	Chromosome 20 open reading frame 26	150962	26074	0.35
HLA-DQA1	Major histocompatibility complex, class II, DQ alpha 1	170258	3117	−1.04
LMOD2	Leiomodin 2 (cardiac)	187222	442721	−0.80
APLP2	Amyloid beta (A4) precursor-like protein 2	223157	334	−0.74
GPR97	G protein-coupled receptor 97	112591	222487	−0.61
FBXO17	F-box protein 17	163400	115290	−0.46
TMEM132B	Transmembrane protein 132B	167812	114795	−0.44
PTK2	Protein-tyrosine kinase 2 (FAK)	128624	5747	−0.44
OSBP2	Oxysterol-binding protein 2	148957	23762	−0.43
C4orf22	Chromosome 4 open reading frame 22	164838	255119	−0.39
ABCG1	ATP-binding cassette, sub-family G (WHITE), member 1	210662	9619	−0.39

a Complete lists of differentially expressed genes (p≤0.01) are given in Supplemental Table S3.

**Abbreviations:** AA: arachidonic acid, ABI: Applied Biosystems, EPA: eicosapentaenoic acid.

**Table 6:** Top 10 up- and down-regulated genes associated with total n-6/n-3 ratio (p≤0.01)^a^.

Gene symbol	Gene name	ABI probe ID	Entrez gene ID	Mean difference
SERPINB9	Serpin peptidase inhibitor, clade B (ovalbumin), member 9 (PI9)	113696	5272	0.57
MSMO1	Methylsterol monooxygenase 1 (SC4MOL)	157577	6307	0.56
SNAP23	Synaptosomal-associated protein, 23 kD	188605	8773	0.55
CACNB1	Calcium channel, voltage-dependent, beta 1 subunit	206077	782	0.51
SLC30A5	Solute carrier family 30 (zinc transporter), member 5	209725	64924	0.51
ISLR	Immunoglobulin superfamily containing leucine-rich repeat	103786	3671	0.48
CCDC78	Coiled-coil domain containing 78	171388	124093	0.48
ACOT13	Acyl-coenzyme A thioesterase 13 (THEM2)	137024	55856	0.46
C12orf43	Chromosome 12 open reading frame 43	230584	64897	0.46
DHX34	DEAH (Asp-Glu-Ala-His) box polypeptide 34	210135	9704	0.45
PRAC	Prostate cancer susceptibility candidate (C17orf92)	177570	84366	−0.64
HGS	Hepatocyte growth factor-regulated tyrosine kinase substrate	118384	9146	−0.62
USP5	Ubiquitin specific protease 5 (isopeptidase T)	195536	8078	−0.60
PLA2G6	Phospholipase A2, group VI (cytosolic, calcium-independent)	149260	8398	−0.55
MRPL55	Mitochondrial ribosomal protein L55	216047	128308	−0.55
SCARF1	Scavenger receptor class F member 1	172281	8578	−0.54
OSBP2	Oxysterol-binding protein 2	148957	23762	−0.50
ASCC2	Activating signal cointegrator 1 complex subunit 2 (p100)	186213	84164	−0.50
NELF	Nasal embryonic luteinizing hormone-releasing hormone factor	159936	26012	−0.47
ABCG1	ATP-binding cassette, sub-family G (WHITE), member 1	210662	9619	−0.47

a Complete lists of differentially expressed genes (p≤0.01) are given in Supplemental Table S4.

**Abbreviations:** ABI: Applied Biosystems.

**Table 7:** Significant overlaps between differentially expressed genes and reported gene sets in MSigDB^a^.

FA ratio	Gene Set Name	K	k	k/K
LA/ALA				
	KRCTCNNNNMANAGC Unknown	60	6	10%
	Immune response	232	11	5%
	Reactome Packaging of telomere ends	49	5	10%
	Reactome Telomere maintenance	77	6	8%
	Immune system process	326	12	4%
	Kegg nitrogen emtabolism	23	3	13%
	RYTAAWNNNTGAY Unknown	53	4	8%
	Locomotory behavior	91	5	5%
	Positive regulation of defence response	10	2	20%
	Response to external stimulus	306	10	3%
	Reactome RNA Pol I promoter opening	59	4	7%
	Reactome DNA strand elongation	31	3	10%
	Hydrolase activity acting on ester bonds	264	9	3%
	Reactome Unwinding of DNA	11	2	18%
	Cofactor transport	11	2	18%
	Reactome Signal attenuation	11	2	18%
AA/EPA				
	Phospholipid transporter activity	12	2	17%
	Lipid transporter activity	27	2	7%
	Nuclear body	33	2	6%
	TCTAGAG, MIR-517	37	2	5%
	Kegg ABC Transporters	44	2	5%
Total n-6/n-3				
	Biocarta Mitochondria pathway	21	2	14%
	Reactome Intrinsic pathway for apoptosis	29	3	4%
	Apoptotic program	60	2	10%
	CCCAGAG,MIR-326	135	4	3%
	SCGGAAGY_V$ELK1_02	822	6	2%
	Post Golgi vesicle mediated transport	14	2	7%
	Intracellular transport	271	5	2%

a The table is sorted by p-values with the lowest first, all p-values<0.01. Number of genes submitted for comparison was 184 for LA/ALA, 36 for AA/EPA, and 65 for n-6/n-3. Gene sets significant at p<0.05 are provided in Supplemental Tables S5–S7.

**Abbreviations:** AA: arachidonic acid, ALA: alpha-linolenic acid, EPA: eicosapentaenoic acid, FA: fatty acid, K: number of genes in gene set, k: number of genes in overlap, LA: linoleic acid.

**Table 8:** Gene Set Enrichment Analysis.

FA ratio^a^	Geneset	Size	Title	NES	NOM p	FDR q
LA/ALA						
	BP00117	26	Jak-STAT cascade	1.79	0.01	0.23
	GO_0006913	30	Nucleocytoplasmic transport	−1.80	0.01	0.16
	GO_0051169	30	Nuclear transport	−1.80	0.01	0.16
	GO_0051188	25	Cofactor biosynthetic process	−1.61	0.03	0.22
	GO_0051168	17	Nuclear export	−1.61	0.03	0.24
AA/EPA						
	BP00101	19	Sulfur metabolism	−2.07	<0.01	<0.01

a There were no significantly enriched gene sets related to n-6/n-3.

**Abbreviations:** AA: arachidonic acid, ALA: alpha-linolenic acid, EPA: eicosapentaenoic acid, FA: fatty acid, FDR: false discovery rate, LA: linoleic acid, NES: normalized enrichment score, NOM p: nominal p-value.

When comparing the highest versus the lowest AA/EPA deciles, differentially expressed genes included the PPARγ target ABCG1 (Table 5 and Supplemental Table S3). Very few inflammatory genes were associated with the AA/EPA ratio. The list of differentially expressed genes overlapped with 24 gene sets mainly related to catabolic processes and fat metabolism, including phospholipid transport (17% overlap), and targets of transcription factors like LXRA/NR1H3 and RXRB (Table 7 and Supplemental Table S6). Seven gene sets that included targets of specific miRNAs were significant, including MIR-103/107 and MIR-7. GSEA revealed significant negative enrichment for sulfur metabolism in the highest AA/EPA decile (Table 8).

Differentially expressed genes resulting from the comparison of the highest versus the lowest total n-6/n-3 deciles included ABCG1, OSBP2 and PLA2, all involved in FA and lipid metabolism (Table 6 and Supplemental Table S4). The list of differentially expressed genes overlapped up to 14% with gene sets related to endoplasmic reticulum and Golgi function, vesicle transport and protein folding (Table 7 and Supplemental Table S7). Apoptotic gene sets were identified, as well as two miRNA target sets: MIR-326 and MIR-296. In addition, differentially expressed genes overlapped with two gene sets sharing promoter motifs that did not match any known transcription factors. According to GSEA, no pathways were significantly enriched according to total n-6/n-3 ratio.

## Discussion

In this explorative analysis, we investigated LA/ALA, AA/EPA and total n-6/n-3 ratios measured in plasma, and their potential effect on blood gene expression in a cross-section of middle-aged Norwegian women. Despite the high consumption of fish and n-3-rich fish oil supplements among Norwegians [11], [12], the population mean of all FA ratios was dominated by n-6 PUFAs. However, the AA/EPA ratio in the lowest AA/EPA and total n-6/n-3 deciles may be regarded as “balanced”, and reflects the frequent use of EPA-rich n-3 supplements in these groups.

When comparing the highest versus the lowest FA ratio deciles, genes and pathways related to inflammation were differentially expressed in all three considered FA ratios. In addition, the identification of genes related to lipid metabolism and autophagy may point to the importance of these processes in elucidating the molecular mechanism related to the differing health effects of n-6 versus n-3 FAs. In general, FAs may influence gene expression profiles via several routes. These include direct mechanisms, such as binding to nuclear transcription factors, but also indirect mechanisms such as production of IP_3_ and DAG. These second messengers lead to modulation of cellular functions and secretion of signaling molecules including eicosanoids. Eicosanoids and related compounds act in a paracrine manner, and may influence the expression profiles of adjacent cells. The gene expression profiles presented here must be viewed as a combined read-out of these mechanisms in blood cells.

### FA Ratios and Inflammatory Signaling

Molecular and epidemiological studies have demonstrated the immunoregulatory effects of dietary FAs. In the present study, the LA/ALA ratio was associated with the largest number of differentially expressed genes compared to the other ratios, both for all genes combined, and regarding genes involved in inflammatory processes. At the pathway level, GSEA revealed that the pleiotropic Jak/STAT pathway was enriched in the highest LA/ALA decile. This pathway is regarded as a principal signaling cascade for numerous cytokines, and its enrichment in the highest LA/ALA decile may be related to the higher level of expression of several cytokines, chemokines, and corresponding receptors and activators in the same group (including IL1R2, IL10RB, CCL7, TLR8). Also, several pathways related to cytokines were among the overlapping gene sets reported in MSigDB. In line with the present findings in a free-living population that consumes high amounts of fish and n-3 supplements, several studies have reported that long-term fish oil supplementation may reduce cytokine production [18], with a potential benefit for inflammatory disease pathogenesis.

A key pro-inflammatory component of innate and adaptive immunity, TLR8, was up-regulated in the highest LA/ALA decile. Studies have shown that dietary n-3 FAs inhibit TLRs, with positive consequences for inflammatory and immune responses [32], [33]. Furthermore, the expression level of TLR8 correlated with poor outcome and increased inflammation in ischemic stroke [34]. TLR8 is highly expressed in peripheral blood leukocytes, and upon activation leads to NF-kB activation and secretion of cytokines. In conjunction with the higher expression levels of cytokines and related factors in the highest LA/ALA decile, TLR8 expression level may be indicative of higher inflammatory status in this group. In support of this hypothesis, the receptor for the pro-inflammatory platelet activating factor, PTAFR, was up-regulated in the highest LA/ALA decile. PTAFR is known to trigger inflammatory and thrombotic cascades, and is important for development of cardiovascular disease. In a dietary intervention study, Ambring *et al*. [13] found reduced numbers of platelets after reducing the n-6/n-3 ratio, which may be mirrored in our data as lower PTAFR expression levels in the lowest LA/ALA decile. The Fc receptor fragments FCGRT and FCER1B (also known as MS4A2), as well as GTF2I, which is necessary for Fc heavy chain transcription, were up-regulated in the highest LA/ALA decile in our study. These fragments are part of the receptors for IgG and IgE, respectively, which are essential mediators of hypersensitive reactions. Conversely, the lower expression levels in the group with favorable LA/ALA ratios, may be viewed as an indication of lower immunological activation associated with lower ratio. The higher expression level of SRGN in the highest LA/ALA decile may also be indicative of hypersensitivity, as this protein is secreted from mast cell granules upon activation. Furthermore, the granzyme B inhibitor SERPINB9 was more highly expressed in the lowest total n-6/n-3 decile, fitting with a reduced immunological activity in the lowest FA ratio deciles.

A common denominator for the processes described above is the relation to hypersensitive reactions in people with conditions like asthma and allergies. Allergen binding to IgE and interaction with the Fc receptor ultimately leads to a release of eicosanoids, platelet activating factor, and cytokines like IL1. Collectively these mediators lead to activation and recruitment of inflammatory cells, which may be related to the increased expression of the chemotactic CCL7 (in the highest LA/ALA decile) and CCL24 (in the highest AA/EPA decile), and the cell migration-related PLAUR (in the highest LA/ALA decile). Several of the identified pathway-level results support this hypothesis. In contrast, we found that a subunit of the antigen presenting MHC II complex, HLA-DQA1, was down-regulated in the highest AA/EPA decile. The molecular mechanisms described here in relation to immunoregluation by FAs, may be a part of the etiology of chronic inflammatory diseases, as suggested by others [35]. Recently, attention has been drawn to asthma and other atopic conditions as risk factors for chronic inflammatory diseases like diabetes and coronary heart disease [36]. The mechanisms of this association remain unclear, but immunological dysregulation is emerging as a potential link between atopy and chronic diseases [37]. Diet is a modifiable risk factor for several chronic diseases, and the gene expression patterns described here may constitute part of the complex and multifactorial links between diet, chronic inflammatory diseases, and atopic conditions.

Contrary to our prior hypothesis, few genes related to inflammatory signaling pathways were differentially expressed according to AA/EPA ratio. AA and EPA are precursors of a variety of inflammatory signaling molecules, and are fundamental to the implication of dietary PUFAs in inflammation [1]. However, in line with our results on gene expression in circulating blood cells, a recent review could make no firm conclusions about marine n-3 PUFAs and circulating inflammatory markers in healthy individuals, or in individuals with risk factors for cardiovascular disease [38].

### The PPARγ Pathway may be Modulated by FA Ratios

Several genes related to lipid metabolism were differentially expressed according to FA ratios, and this was reflected in the pathway level analysis as well. Significant overlaps with reported gene sets from MSigDB included phospholipid transporter activity and Kegg ABC transporters (associated with the AA/EPA ratio), as well as post Golgi vesicle mediated transport and intracellular transport (associated with the total n-6/n-3 ratio). According to LA/ALA ratio, no pathway level processes were significant, but key FA metabolism regulators like RXRA, FABP4 (both binding partners for PPARγ), and ADIPOR1 were differentially expressed. These key regulators have been implicated in the pathogenesis of atherosclerosis, diabetes and metabolic diseases [39]–[41]. The PPARγ target ABCG1 was one of very few genes that were significantly associated with more than one FA ratio: it was expressed at lower levels in the highest AA/EPA and n-6/n-3 deciles. ABCG1 promotes cholesterol efflux from macrophages, and reduced expression in diabetics was related to increased foam cell formation [42]. The association of low ABCG1 levels with higher FA ratios in our dataset may provide hints to important atherogenic consequences of ratios dominated by n-6 FAs. Taken together, one may speculate that the PPARγ pathway is modulated by differing FA ratios, and that possible health gains may be achieved via this pathway, by lowering FA ratios that are dominated by n-6 FAs.

### FA Ratios may Influence Autophagy

Unexpectedly, several genes related to autophagy were expressed at higher levels in the lowest deciles of all FA ratios. Key genes included the positive regulator KIAA0831 (also known as Barkor/Atg14, up-regulated in lowest LA/ALA) and OPTN (also known as FIP2, up-regulated in the lowest LA/ALA and total n-6/n-3 deciles). Autophagy is emerging as a highly specific and diverse mechanism for lysosomal degradation of intracellular material, with numerous implications for cellular homeostasis, nutrient sensing, insulin regulation, and cholesterol metabolism [43]. Data is accumulating that demonstrates the close link between lipid metabolism and autophagy: lipid droplets may be degraded by autophagy, modulation of membrane lipids is essential for the process, and lipid modifying proteins have important regulatory roles [44]. Related processes include the inositol phosphate signaling system, endoplasmic reticulum stress, and oxysterol metabolism, all of which were reflected in our gene expression data. In line with our findings related to lipid metabolism and autophagy markers, a very recent cell line study showed induction of autophagy after n-3 FA supplementation, via the PPARγ pathway [45]. The potential influence of FA ratios on autophagy, and the impact of autophagy on health and disease, warrants further study.

### miRNAs and unknown Transcription Factor Motifs

Differentially expressed genes derived from all FA ratios significantly overlapped with one or more gene sets that included targets of specific miRNAs. For example, miRNAs related to AA/EPA ratio included MIR-103/−107 and MIR-7, both shown to be involved in insulin and insulin-like growth factor signaling [46], [47]. Total n-6/n-3 ratio was associated with MIR-326, which is involved in autoimmune diseases [48], and MIR-296, which is involved in regulation of angiogenesis [49] and lipoapoptosis [50]. Transcriptional regulation by miRNAs may emerge as a mechanism for fine-tuning cellular and physiological responses to FAs. Furthermore, gene expression profiles according to LA/ALA and total n-6/n-3 ratios overlapped with gene sets containing common promoter motifs matching unknown transcription factors. One could speculate that transcription factors as yet unidentified may play a role in the complex transcriptional regulation in response to dietary lipids.

### Strengths and Limitations

Because the Norwegian population consumes comparably high amounts of n-3 PUFAs, they constitute a “natural experiment” for exploring the potential effects of FA ratios. However, several issues arise in the interface between epidemiologic and molecular research. First, as this is an explorative, cross-sectional study, no conclusions can be made regarding causal mechanisms. Importantly, residual bias may be present in the data set, for example related to age, ongoing infections, or the variation in time since previous meal before blood sample collection.

There was a small, but statistically significant age difference between women in the highest versus lowest AA/EPA and total n-6/n-3 deciles. This was likely related to the finding that n-3 supplement users were slightly older than non-users in the study population. Age is known to influence metabolism of n-3 PUFAs, particularly during menopause. However, due to the use of supplements, the small age differences (3 years for AA/EPA ratio, and 4 years for total n-6/n-3 ratio), and our confirmed data on menopausal status, we chose not to adjust for age. This may have introduced bias in the data. Additional bias may have been introduced by confounders on which we had either limited or no information. For example, disease status could only be inferred from self-reported medication use, and no data on the distribution of blood cell subtypes were available. Because the PAXgene Blood RNA kit preserves RNA from all circulating cells, fluctuating levels of blood cell subtypes related to an infection may influence gene expression profiles. The use of medication was not statistically different between comparison groups, but sub-clinical infections could still introduce differences in blood cell subtypes, and in turn, in the gene expression profiles.

In our study design, study women were not asked to fast before blood collection. Humans spend the majority of the day in a non-fasted, postprandial state, with a continuous fluctuation in the degree of lipemia [51]. Our biomarker of exposure, plasma total FAs, includes both the highly variable triglycerides (varies within hours and reflects FA content of the previous meal [52]), the cholesterol esters and phospholipids (varies within days) and the more stable non-esterified (free) FAs [53]. Thus, this biomarker may be said to reflect both intake and metabolism, and has been judged a valid biomarker, particularly for the exogenously derived long-chain PUFAs [53]. However, this biomarker renders us unable to differentiate between short- versus long-term exposure to FAs. Studies have shown differing gene expression profiles after short (postprandial) and long-term exposure to n-3 FAs. Postprandially, genes involved in lipid metabolism were regulated [19], and after long-term exposure inflammatory signaling was regulated [5]. In line with the properties of plasma FA ratios as a biomarker of exposure, we identified gene expression differences related to both lipid metabolism and inflammation.

Regarding the data analysis, GSEA can only identify gene sets that are being used as input, in this case pathways from KEGG, GO, and Panther. Recently characterized pathways like autophagy may be reported to involve somewhat non-overlapping sets of genes according to different databases, which poses difficulties for GSEA and overlap analysis. Lastly, we did not validate gene expression levels using other methods like quantitative real-time polymerase chain reaction or independent data sets, and our results must be interpreted accordingly.

### Conclusions

We conclude that the plasma ratios of LA/ALA, AA/EPA and total n-6/n-3 may have differential impacts on blood gene expression in this cross-section of Norwegian middle-aged women. Genes and pathways associated with differing FA ratios may provide links to potential health consequences related to the balance of n-6 and n-3 PUFAs. Increased knowledge and awareness of the metabolic actions of dietary PUFAs is warranted, in order to form a sound basis on which to deliver evidence-based nutritional advice to the public.

## Supporting Information

Figure S1 **Venn diagram showing the overlap of differentially expressed genes by LA/ALA, AA/EPA and total n-6/n-3.** Abbreviations: AA: arachidonic acid, ALA: alpha-linolenic acid, EPA: eicosapentaenoic acid, LA: linoleic acid.
(TIFF)

Table S1 **Additional characteristics of the study population (n = 227) and comparison groups (group n = 23).**
(XLSX)

Table S2 **Differentially expressed genes according to LA/ALA ratio (t-tests, p<0.01).**
(XLSX)

Table S3 **Differentially expressed genes according to AA/EPA ratio (t-tests, p<0.01).**
(XLSX)

Table S4 **Differentially expressed genes according to total n-6/n-3 ratio (t-tests, p<0.01).**
(XLSX)

Table S5 **MsigDB gene set overlap based on LA/ALA ratio (p<0.05).**
(XLSX)

Table S6 **MsigDB gene set overlap based on AA/EPA ratio (p<0.05).**
(XLSX)

Table S7 **MsigDB gene set overlap based on total n-6/n-3 ratio (p<0.05).**
(XLSX)
