# Supplementary figures and images for: Plasma Fatty Acid Ratios Affect Blood Gene Expression Profiles - A Cross-Sectional Study of the Norwegian Women and Cancer Post-Genome Cohort
Source: PLoS One. 2013 Jun 25;8(6):e67270. doi: 10.1371/journal.pone.0067270 (PMC3692510; doi:10.1371/journal.pone.0067270)

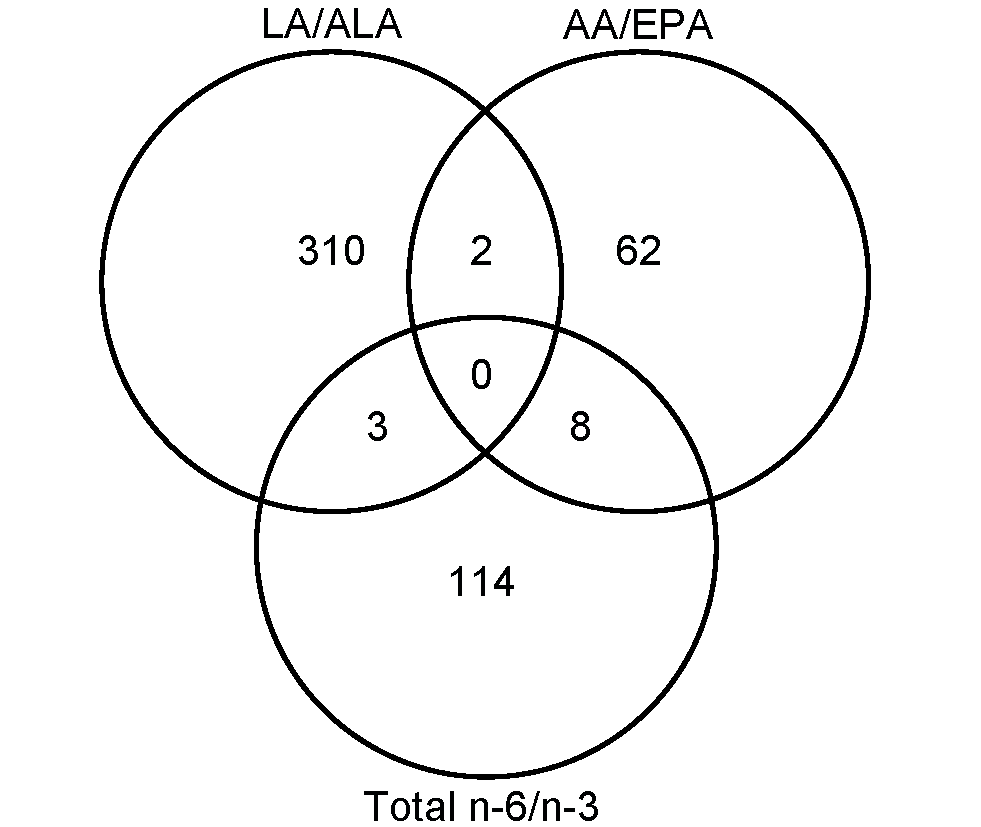

Supplement: Figure S1 — Venn diagram showing the overlap of differentially expressed genes by LA/ALA, AA/EPA and total n-6/n-3. Abbreviations: AA: arachidonic acid, ALA: alpha-linolenic acid, EPA: eicosapentaenoic acid, LA: linoleic acid. (TIFF) [file pone.0067270.s001.tiff]
